# Dietary Patterns and Chronic Disease Risk: A Proteomic Framework to Strengthen Causal Inference^☆^

**DOI:** 10.1016/j.advnut.2026.100755

**Published:** 2026-10-13

**Authors:** Rob M van Dam, Charlie GY Lim, Marilyn C Cornelis

**Affiliations:** 1 Department of Exercise and Nutrition Sciences, Milken Institute School of Public Health, The George Washington University, Washington, DC, United States; 2 Saw Swee Hock School of Public Health, National University of Singapore, and National University Health System, Singapore, Republic of Singapore; 3 Department of Preventive Medicine, Northwestern University Feinberg School of Medicine, Chicago, IL, United States

**Keywords:** nutrition, dietary pattern, proteomics, biomarkers, Mendelian randomization, epidemiology

## Abstract

The strength of evidence in nutrition research is hampered by the difficulty of maintaining long-term dietary compliance in randomized trials. Despite providing valuable evidence, short-term feeding trials typically rely on a limited set of surrogate outcomes, and observational studies are susceptible to measurement error and residual confounding. We propose a complementary research framework that integrates randomized feeding trials with Mendelian randomization (MR) using proteomic markers to strengthen causal inference in nutrition research. In this approach, feeding trials are used to identify proteins that respond to dietary patterns, and MR analyses are subsequently used to assess whether these diet-responsive proteins are relevant to the development of chronic diseases. In addition to instrumenting individual proteins, we propose using genetic scores to instrument proteomic signatures of dietary patterns in MR analyses. We argue that proteins are particularly informative biomarkers because they reflect multiple biological pathways involved in disease pathophysiology and can often be instrumented with *cis-*protein quantitative trait loci, which reduces concerns about horizontal pleiotropy compared with other MR approaches. To illustrate the feasibility of several steps in the proposed approach, we highlight studies of the Dietary Approaches to Stop Hypertension diet. This research shows that randomized feeding trials can identify reproducible proteomic changes and that several of these associations can be confirmed in population-based cohort studies. We further note that selected diet-related proteins have been associated with risk of type 2 diabetes in prospective studies and, in some cases, supported by MR and colocalization analyses. We also discuss potential limitations, including incomplete proteomic coverage, measurement error, epitope effects, and challenges in satisfying MR assumptions. In summary, we argue that a cross-design, proteomic-based research strategy can contribute to the triangulation of evidence in nutrition research, generating more specific and robust dietary recommendations for chronic disease prevention.

**Statement of significance**

Mendelian randomization (MR) has been used in nutrition research, but prior research has not integrated evidence from randomized feeding trials and cohort studies with proteomic MR analyses. This Perspective proposes a novel strategy that uses randomized trials to identify diet-responsive proteins and applies MR to test whether those proteins affect chronic disease risk, providing a strategy to strengthen evidence on the effects of dietary patterns on chronic disease risk.

## Background

Dietary risk factors are among the most critical determinants of chronic disease and premature mortality worldwide [1]. However, research on the role of dietary factors in the development of chronic diseases faces several methodological challenges. Double-blind, placebo-controlled, randomized trials with disease outcomes are generally regarded as the strongest source of evidence in medical research. These types of studies have been conducted for food components such as vitamins, minerals, or extracts that can be administered in capsule form [2]. However, evaluating dietary patterns in randomized trials has been more challenging because adherence to dietary advice typically declines over several years. For example, in the Women’s Health Initiative Dietary Modification Trial, which included a high-fruit-and-vegetable, low-fat diet, the intervention and control group diets converged over time [3]. In the PREDIMED study (Prevención con Dieta Mediterránea), which evaluated a Mediterranean diet, compliance with consumption of freely provided foods (i.e., nuts and extra-virgin olive oil) appeared better than with dietary advice [4], but this approach is costly in large-scale studies. Given the challenges of long-term trials, much of the evidence on the impact of dietary patterns on disease risk is based on a combination of prospective cohort studies and shorter-term trials that use disease biomarkers as outcomes.

Until recently, feeding trials relied on a limited set of disease biomarkers, such as blood lipids, glycemic markers, and inflammatory markers, as outcomes. Moreover, several commonly used biomarkers, such as C-reactive protein [5] and HDL cholesterol [6], may not be causally related to disease development, making it unclear whether effects on these biomarkers translate into an impact on disease outcomes. Proteomic markers can substantially expand this set of biomarkers, possibly reflecting a broader range of biological pathways relevant to disease development [7], [8]. Prospective cohort studies directly relating dietary patterns to chronic disease outcomes do not rely on disease biomarkers. However, cohort studies may be affected by loss to follow-up, measurement error, and residual confounding. Triangulation, the convergence of evidence from various research approaches with different methodological limitations, provides the strongest evidence for the causal effects of dietary factors on disease risk [9].

Mendelian randomization (MR) is a research method based on the principle that genes are randomly assorted from parents to offspring during gamete formation and conception [10]. MR uses genetic variants as instrumental variables (proxies) of modifiable exposures. Because germline variants are fixed at conception, MR can reduce confounding by nongenetic factors and reverse causation. Protein concentrations may be particularly suitable for MR analysis, as genetic variants can directly affect protein abundance [11]. In this perspective paper, we propose a complementary research approach integrating evidence from randomized feeding trials and MR using proteomics to strengthen research on dietary patterns and chronic disease risk. In this framework, randomized trials identify diet-responsive proteins, and MR evaluates whether these proteins are relevant for the development of chronic diseases. We do not aim to quantify the effect of dietary patterns on chronic disease risk using our proteomic-based approach. Instead, we aim to assess the evidence for the effects of dietary patterns on chronic disease risk through various biological pathways, as reflected in protein levels. The results can contribute evidence for causal effects of dietary patterns on chronic disease risk in the context of triangulation with other types of research.

## Progress in Measuring Proteomic Profiles and Relating These to Chronic Disease Risk

Proteomics is valuable for studies of determinants of chronic disease risk, as it includes proteins involved in numerous biological pathways and can identify potential targets for preventive interventions [12]. Advances in laboratory measurements enable the large-scale measurement of thousands of proteins using Olink antibodies, SomaScan aptamers, mass spectrometry, or other assays [13]. Large-scale proteomic data are now available for several prospective cohort studies worldwide [14]. Proteogenomic data allow identification of genetic variants that reflect variation in protein concentrations [15]. These genetic variants can subsequently be used as instrumental variables for proteins in MR analyses of chronic disease outcomes (Figure 1). Instrumental variables in MR must satisfy 3 assumptions [10]. First, the instruments are associated with the exposure (relevance). Second, the instruments do not share a common cause with the outcome (independence). Third, the instruments affect the outcome only via the exposure (exclusion restriction). Genetic variants should not affect the outcome through biological pathways independent of the exposure of interest (i.e., horizontal pleiotropy). For proteins, 2 types of genetic instruments can be identified: *cis-*protein quantitative trait loci (pQTLs) and *trans*-pQTLs [11]. *Cis*-pQTLs are genetic variants located near the gene encoding the protein, and *trans*-pQTLs are genetic variants located farther from that gene. *Cis*-pQTLs are less likely than *trans*-pQTLs to affect biological pathways independent of the protein [11]. As a result, using *cis*-pQTLs in MR may reduce the risk of horizontal pleiotropy. Multiple proteins have been associated with chronic disease risk in prospective cohort studies, and several have been confirmed in MR analyses [7], [16]. Thus, high-throughput affinity-based proteomic platforms hold promise for identifying novel targets for disease development. Although proteomic platform technology is rapidly improving, coverage remains selective and can be affected by measurement error as described in Potential Limitations of Proteomic Approaches in Nutrition Research section [13].

**FIGURE 1:**
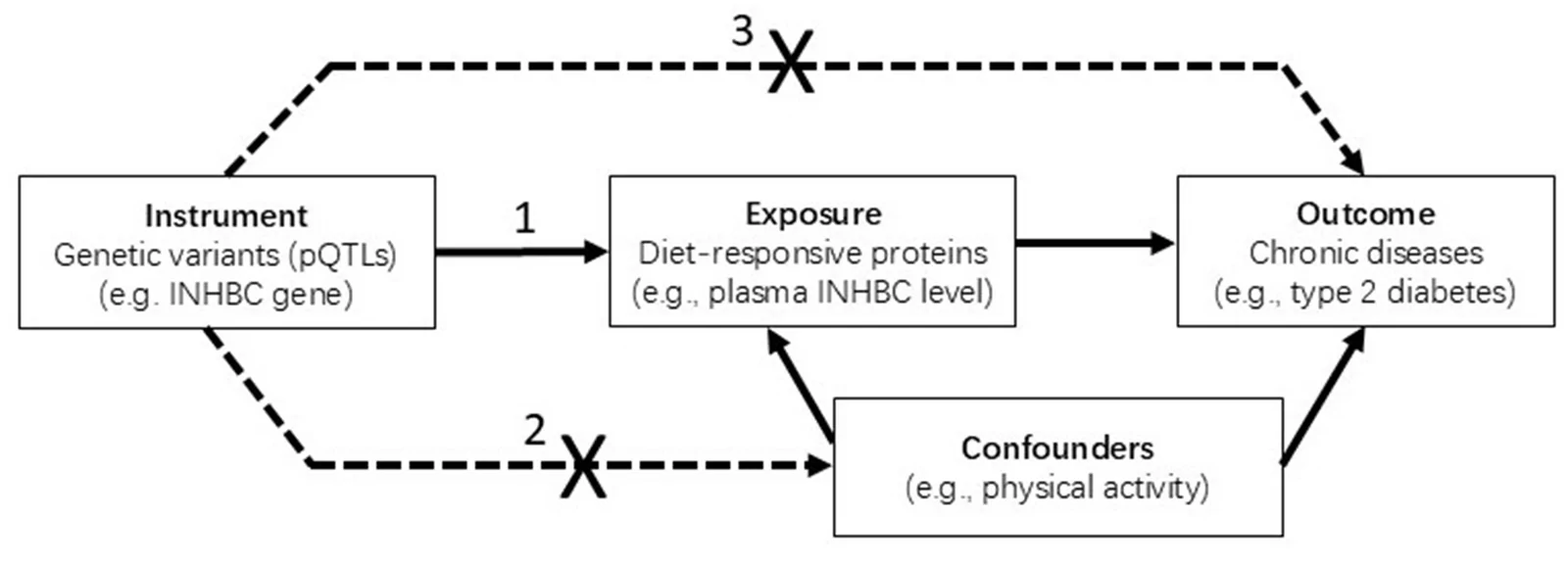
Mendelian randomization to assess the effect of diet-responsive proteins on chronic disease outcomes using genetic instruments. The genetic variant serves as a “proxy” for the protein, and the protein’s effect on chronic disease is assessed based on the association of the genetic variant with the protein and with chronic disease. The numbers refer to the assumptions made: *1*) Relevance: the genetic variant used as an instrument is robustly associated with the protein of interest; *2*) Independence: the genetic variant is independent of confounders that influence both the protein and chronic disease outcome; *3*) Exclusion restriction: the genetic variant affects the outcome only through its effect on the protein, not through any alternative pathway [10]. The example uses the DASH diet-responsive INHBC protein and type 2 diabetes. INHBC, Inhibin β C chain; pQTL, protein quantitative trait loci.

## Proteomic Markers of Dietary Patterns

Evidence is rapidly accumulating that dietary patterns are robustly associated with proteomic markers. Various dietary patterns, including the Alternative Healthy Eating Index (AHEI), the Healthy Eating Index, Mediterranean Diet Scores, and the Dietary Approaches to Stop Hypertension (DASH) score, have been evaluated in relation to proteomic markers [17], [18], [19], [20], [21], [22], [23]. Multiple proteins were robustly associated with dietary patterns, with these associations confirmed across different study populations [20], [21]. For example, we identified proteins consistently associated with AHEI and DASH scores in Black adults in the United States and Asian adults in Singapore [21]. Biological pathways associated with healthy dietary patterns based on protein associations included complement and coagulation cascades, cell adhesion molecules, and lysosome pathways [17], [20]. In addition to individual proteins, proteomic signature scores have been derived to reflect a range of chronic disease risk factors [24]. Zhu et al. [17] derived proteomic signatures for healthy dietary patterns in the United Kingdom Biobank and showed that these were associated with lower risks of type 2 diabetes (T2D), cardiovascular diseases, chronic respiratory and kidney diseases, and cancers. They also estimated that circulating proteins may mediate a substantial proportion of the association between dietary patterns and these diseases. Other studies have identified plasma proteins that are potential mediators of the effects of dietary patterns on the development of dementia [25], [26]. These proteomic marker findings can point to biological pathways linking dietary patterns to chronic disease risk. Still, observational studies of dietary patterns and proteomic markers can be affected by residual confounding and reverse causation, making it difficult to establish that dietary patterns per se caused differences in protein levels. We therefore recommend using randomized feeding trials in the proposed framework to more directly assess the effect of dietary patterns on protein levels.

## A Proposal for a Research Strategy Integrating Proteomics into Studies of Dietary Patterns and Chronic Disease Risk

We propose a cross-design integrative framework that combines data from feeding trials and MR analysis using proteomics, a “randomized controlled trial (RCT)-MR analysis” (Figure 2). First, a randomized feeding trial is used to evaluate the effects of dietary factors on proteomic markers that reflect preclinical pathophysiological changes. Second, genetic variants (pQTLs) associated with these diet-related proteomic markers are identified. Third, these genetic variants are examined in relation to chronic disease outcomes using MR analysis (Figure 1). The second step can be conducted using existing genome-wide association studies (GWAS) of proteomic markers, and the third step can use summary data from GWAS disease consortia. A similar approach combining a randomized trial with MR has previously been used in research on metabolomic biomarkers and prostate cancer [27].

**FIGURE 2:**
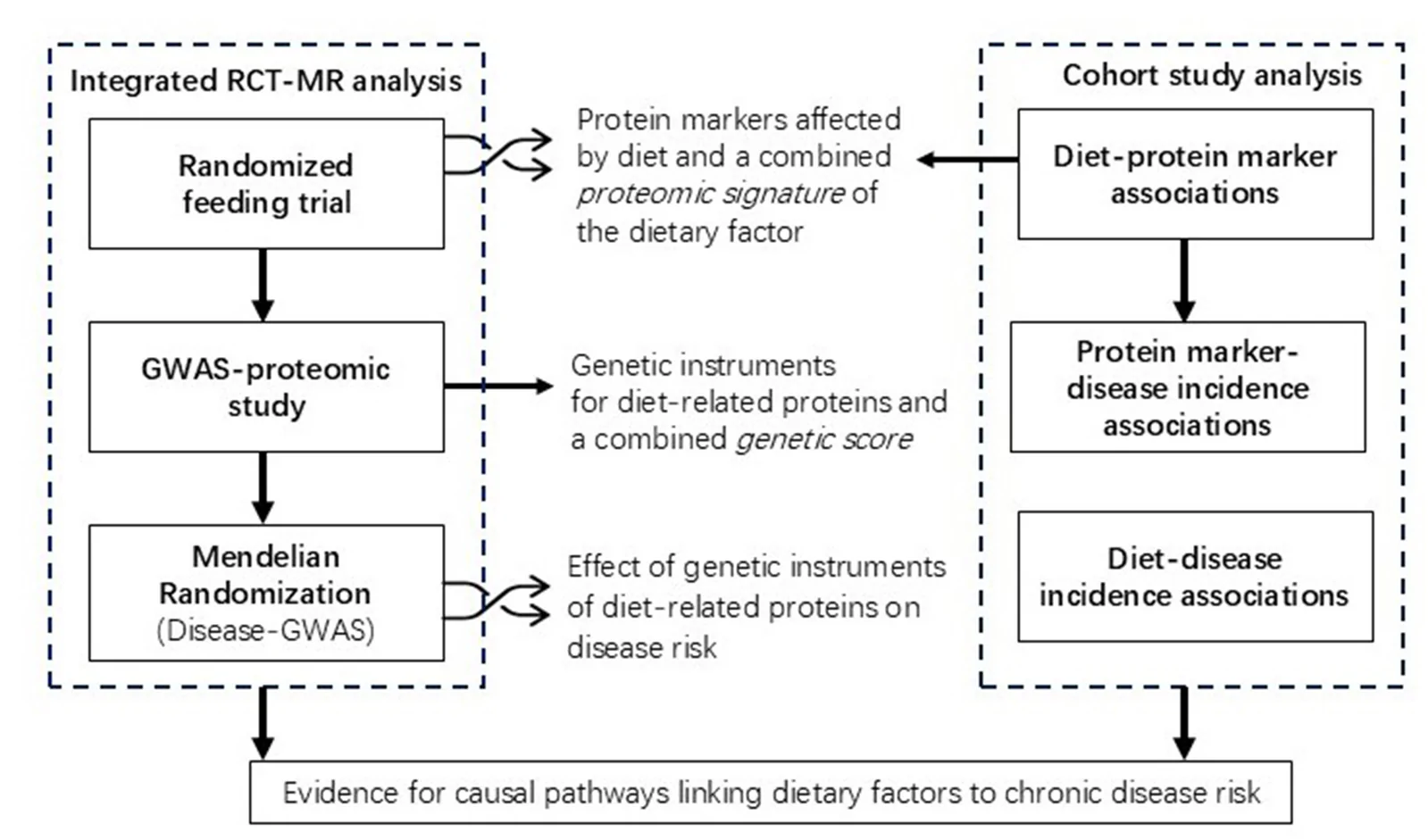
A research strategy integrating randomized feeding trials and Mendelian randomization (RCT-MR analysis) with complementary conventional cohort analyses to strengthen evidence of causal effects of dietary factors on chronic disease risk using proteomic markers. The proteomic signature is a weighted combination of measured protein concentrations associated with the dietary factor. The genetic score is a weighted combination of alleles that predicts the proteomic signature. GWAS, genome-wide association study; RCT-MR, randomized controlled trial-Mendelian randomization.

This RCT-MR analysis approach reduces several sources of confounding and bias. Randomized feeding trials minimize confounding. Furthermore, using biomarkers rather than disease incidence as the outcome allows for a shorter trial duration and a more intensive intervention, ensuring high dietary compliance. Using genetic instruments in MR minimizes confounding by modifiable risk factors and reverse causation [10]. Moreover, using proteins as biomarkers enables the identification of genetic variants closely linked to protein expression (i.e., *cis*-pQTLs), reducing the likelihood of horizontal pleiotropy in MR analyses [11]. Although *cis*-pQTLs should be prioritized, *trans*-pQTLs affecting proteins encoded by distant genetic variants may be included to improve protein predictions using genetic scores. MR assumptions should be evaluated, and sensitivity analyses, including Egger and weighted MR, as well as colocalization, should be conducted to assess pleiotropy [28]. Furthermore, bidirectional MR can be used to evaluate whether reverse causation (i.e., effects of disease on protein levels) may explain the observed associations [28].

Correlations among proteins should be considered throughout analysis and interpretation. In analyses of diet–proteomic marker associations, tests across proteins are dependent, so multiplicity should be controlled, commonly using false-discovery-rate procedures that account for dependence [29]. When deriving proteomic signatures of dietary patterns, correlated proteins can yield unstable coefficients, and penalized methods such as elastic net can improve stability [30]. In MR analyses, protein-specific *cis*-pQTLs may help distinguish effects of correlated proteins when sufficiently distinct instruments are available [11]. However, shared genetic regulation, linkage disequilibrium, and pleiotropy can complicate interpretation; conditional analyses and colocalization can help assess whether associations reflect distinct or shared genetic signals [28]. Multivariable MR can estimate the direct effect of each protein conditional on correlated proteins, but conditional instrument strength should be evaluated because weak instruments may arise when exposures share similar genetic predictors [31]. Conversely, protein correlations can be informative in pathway-level analyses that integrate signals across proteins to identify broader biological processes associated with dietary patterns [20].

In addition to identifying individual proteins affected by a dietary pattern, a proteomic signature can be identified as a weighted combination of measured protein concentrations associated with the dietary pattern [17]. Subsequently, in a proteomic GWAS, a genetic score can be derived as a weighted combination of genetic variants (i.e., pQTLs) that predict the proteomic signature. In addition to instrumenting individual proteins, we propose using genetic scores to instrument the proteomic signature of a dietary pattern in MR analyses. Because such a score aggregates the genetically predicted levels of multiple diet-responsive proteins, it may increase statistical power to detect an aggregate effect, provided that the constituent proteins act on disease in a consistent direction. In addition, this approach reduces the multiple-testing burden compared with testing each protein individually. The proteomic signature can be treated as a single exposure, with its genetic instruments obtained directly by regressing the signature score on conditionally independent *cis*-pQTLs of its constituent proteins in an independent sample; when individual-level data are unavailable, an equivalent instrument can instead be assembled from published pQTL–protein effect estimates weighted by each protein’s signature loading, with signs preserved so that the score increases with adherence. The genetic score can then be related to chronic disease risk using individual-level (1-sample) or summary-level (2-sample) MR [32]. Alternatively, a single lead *cis*-pQTL per protein can be used as a conservative approach when instrument independence is uncertain. Optimal approaches to using genetic instruments to estimate dietary pattern protein scores, rather than individual protein scores, require further evaluation in future studies.

Ideally, the described approach is complemented by a series of “conventional” epidemiological analyses (Figure 2). Cohort studies of diet and proteomic markers can confirm findings from the randomized feeding trial. Alternatively, these cohort analyses can be done first to prioritize proteins for evaluation in randomized trials, reducing the number of tests and maintaining statistical power in trials with smaller sample sizes. These analyses can be cross-sectional, with diet and proteins measured at the same time point, or longitudinal, with changes in diet related to changes in proteins over the same period. In feeding trials, the provided meals, study population characteristics, and other environmental exposures may vary less than in free-living populations. It is therefore useful to confirm that proteomic markers identified in feeding trials reflect dietary patterns under “real-world” conditions. In addition, observational studies can assess long-term diets, thereby evaluating whether the short-term effects observed in feeding trials extend to long-term effects on protein concentrations. Next, results of analyses of protein concentrations and disease risk in prospective cohort studies can be compared with MR results. Finally, dietary patterns assessed using validated instruments, such as food frequency questionnaires or biomarker-based intake scores, can be directly linked to disease risk. Consistency across analyses based on the proposed cross-design framework and conventional epidemiological analyses represents a triangulation approach that strengthens evidence for causal effects.

## The DASH Dietary Pattern, Proteins, and Diabetes Risk

We will illustrate the feasibility of several steps in the proposed proteomic-based approach, using examples from the DASH dietary pattern and T2D. We will show that RCTs and cohort studies can identify the impact of dietary patterns on protein markers, and that these diet-related proteins can be linked to T2D using conventional and MR analyses. We focus on the DASH diet as a proof of principle because it has been evaluated in relation to proteomic markers in both feeding trials and cohort studies [17], [18], [19], [20], [21], [22], [23]. The DASH diet emphasizes fruits, vegetables, low-fat dairy, whole grains, nuts, and legumes and is low in red and processed meat and sugary foods and beverages [33]. Although it was initially developed to control hypertension, the DASH diet score has been associated with a lower risk of type 2 diabetes, cardiovascular diseases, cancer, and premature mortality in prospective cohort studies [34].

The DASH and DASH-sodium studies were randomized feeding trials [19], and serum protein levels were assessed using SomaScan measurement (∼7000 proteins). Changes in serum proteins were compared between the DASH and control diets, with 8 wk of DASH diet consumption in the DASH trial and 4 wk in the DASH-Sodium trial. In a pooled analysis, the concentrations of 71 proteins changed significantly after false-discovery-rate correction. The DASH diet score has been evaluated in relation to comprehensive proteomic measures in 6 cohorts in the United States, United Kingdom, and Singapore using cross-sectional analyses [17], [18], [19], [20], [21], [22], [23]. Supplemental Table 1 shows the cohort characteristics. Proteomic measurements were conducted using the SomaScan or Olink platforms (∼1300–5300 proteomic markers).

As shown in Table 1 [15], [16], [17], [18], [20], [21], [23], [35], [36], [37], [38], [39], [40], [41], [42], [43], [44], [45], [46], [47], [48], [49], [50], [51], [52], [53], 8 proteins identified in the DASH trials were confirmed in ≥2 cohorts, including epidermal growth factor receptor, afamin (AFM), inhibin β C chain (INHBC), hepatocyte growth factor receptor (MET), C-C motif chemokine 25, β-Ala-His dipeptidase (CNDP1), inhibin β A chain, and retinol-binding protein 4. The SomaScan measurements for these proteins all had *cis*-pQTLs and were confirmed by mass spectrometry or Olink measurements, supporting the accuracy of the measurements (Supplemental Table 2). Twenty-two other proteins were not significant in the DASH trials but were consistently associated with the DASH score in ≥4 cohorts (Supplemental Figure 1). These studies show that robust associations between dietary patterns and proteomic markers can be observed across diverse populations, dietary assessment methods, and proteomic platforms. Moreover, existing studies varied substantially in sample size, the platform used (Olink compared with SomaScan), and the number of evaluated proteomic markers. Thus, the number of confirmed proteins is likely to increase substantially with increasing sample sizes, consistent platforms, and greater protein coverage.

**TABLE 1:** Proteins identified in DASH intervention studies, replication in cohort studies, and associations with T2D

Protein name	Gene symbol	Potential biological functions	Effect direction in DASH trials	Results from cohorts		Summary on DASH diet-T2D risk mediation
				Replicating DASH association^1^	Association with T2D^2^	
Epidermal growth factor receptor	EGFR	EGFR is a tyrosine kinase involved in key signaling pathways regulating cell proliferation, differentiation, survival, adhesion, and migration [35]. EGFR inhibition impeded adipogenesis in human preadipocytes in vitro, suggesting that EGFR plays a role in maintaining adipose tissue function [36].	+	ARIC [20], JHS [21], MEC [21], CRIC [23], FHS [20]	Inverse in FHS/MDCS [37] and MEC [38]	Potential mediator, but MR confirmation required
Afamin	AFM	AFM is a specific binding protein for vitamin E and may be involved in lipid and glucose metabolism [39]. Higher AFM levels have been associated with increased hepatic lipid content, insulin resistance, and reduced insulin sensitivity in humans [40].	_	UKB [17], JHS [21], CRIC [23]	Direct in AGES [16], ARIC [41], FHS/MDCS [37], JHS [42], KORA [43], and MEC [38]. MR analysis suggests a direct effect of T2D on AFM [16].	Bidirectional MR analysis does not support mediation but suggests reverse causation.
Inhibin β C chain	INHBC	INHBC may influence systemic metabolism by activating ALK7 in adipose tissues [44]. Higher plasma INHBC levels have been linked to reduced lower-body fat and atherogenic dyslipidemia, as well as increased systemic inflammation and blood glucose concentrations [44].	_	UKB [17], ARIC [20], CRIC [23]	Direct in AGES [16], ARIC [41], CHS [45], and MEC [38]. MR analyses support a direct effect of INHBC on T2D [38] and a direct effect of T2D on INHBC [16], [42].	Potential mediator with inconsistent MR confirmation
Hepatocyte growth factor receptor	MET	MET is involved in regulating hepatic glucose metabolism, and MET deletion in mice led to diminished β-cell mass, reduced glucose tolerance, and impaired insulin secretion [46], [47], [48].	+	UKB [17], ARIC [20], FHS [18]	Inverse in ARIC [41], CHS [45], and MEC [38]. Genetically predicted MET was associated with T2D in UKB [15].	Potential mediator, but further MR confirmation is required
C-C motif chemokine 25	CCL25	CCL25 is a chemokine involved in immune and inflammatory responses. CCL25 may act via the Gαi-coupled receptor CCR9 to impair β-cell function by inhibiting insulin secretion and promoting cytokine-induced apoptosis [49].	_	ARIC [20], FHS [18]	Direct in ARIC [41], FHS/MDCS [37], JHS [42]	Potential mediator, but MR confirmation required
β-Ala-His dipeptidase	CNDP1	CNDP1 encodes serum carnosinase 1, a dipeptidase that hydrolyzes histidine-containing dipeptides, including carnosine, anserine, and homocarnosine [50].	+	MEC [21], FHS [18]	Direct association in FHS/MDCS [37]	Unlikely to be a mediator based on the suggested direction of effects.
Inhibin β A chain	INHBA	INHBA may modulate insulin secretion in the presence of glucose [51].	_	ARIC [20], CRIC [23]	Direct association in AIRC [41], MEC [38].	Potential mediator, but further MR confirmation required
Retinol-binding protein 4	RBP4	RBP4 mediates retinol transport in blood plasma and directly correlates with plasma retinol concentrations [52].	+	ARIC [20], FHS [18]	Direct association in several cohorts [53]	Unlikely to be a mediator based on the suggested direction of effects.

Abbreviations: AGES, AGES-Reykjavik study; ARIC, Atherosclerosis Risk in Communities; CHS, Cardiovascular Health Study; CRIC, Chronic Renal Insufficiency Cohort; DASH, dietary approaches to stop hypertension; FHS, Framingham Heart Study; FHS/MDCS, meta-analysis of the Framingham Heart Study and the Malmö Diet and Cancer Study; JHS, Jackson Heart Study; MEC, Singapore Multi-Ethnic Cohort; MR, Mendelian randomization; T2D, type 2 diabetes; UKB, United Kingdom Biobank; KORA, Cooperative Health Research in the Region of Augsburg.

1 Directionally consistent and significant associations between the DASH score and blood protein concentrations.

2 Significant associations between blood protein concentrations and incident T2D.

Several of the 8 proteomic markers associated with dietary patterns in the trials and cohort studies have also been linked to T2D in conventional cohort and MR analyses. For 6 of the 8 proteins, associations with T2D incidence were observed in the expected direction in cohort studies (Table 1). We will illustrate MR analysis and its assumptions using INHBC and T2D as an example based on Liang et al. [38] (Figure 1). For the relevance assumption, a single *cis*-pQTL was used to instrument plasma INHBC levels. The F-statistic for this specific instrument was not reported. However, a separate analysis reported a very high F-statistic (>1300) for a *cis*-pQTL, demonstrating that INHBC can be strongly instrumented [54]. Regarding the independence assumption, genetic instruments substantially reduce confounding by lifestyle factors. However, Liang et al. used a multiancestry T2D GWAS. Although the GWAS accounted for population structure, residual population stratification cannot be completely excluded. For the exclusion restriction assumption, use of a *cis*-pQTL reduces but does not eliminate concern about effects through alternative biological pathways (i.e., horizontal pleiotropy). Because only 1 genetic instrument was available for INHBC, multi-instrument sensitivity analyses for pleiotropy could not be performed. Colocalization analysis supported the absence of confounding by distinct causal variants in linkage disequilibrium: the posterior probability that the INHBC pQTL and T2D associations shared the same causal variant was 71.2%. SomaScan INHBC levels correlated well with Olink measurements (Supplemental Table 2). Still, epitope effects from protein-altering variants pose an additional challenge for proteomic MR because a variant may affect affinity-based assay binding or protein structure rather than circulating protein abundance. Finally, causal direction remains uncertain. Previous MR studies have provided evidence that T2D itself may affect circulating INHBC levels (Table 1), raising the possibility of reverse causation. Thus, INHBC is a plausible candidate linking the DASH diet to T2D, but the current evidence does not conclusively establish that circulating INHBC mediates the effect of the DASH diet on T2D risk. Another example is MET, which was associated with greater adherence to the DASH diet and with a lower risk of T2D across several cohorts (Table 1). Genetically predicted MET levels were inversely associated with T2D in the United Kingdom Biobank [15], providing initial support that requires confirmation in an independent MR analysis. In contrast, bidirectional MR results suggest that reverse causation may explain the association between AFM and T2D, arguing against prioritizing AFM as a potential mediator [16]. In Swedish cohorts, MR and colocalization analyses found evidence of causal effects of 4 proteins associated with healthy dietary patterns on T2D development [55]. The DASH diet and diabetes example shows that consistent dietary pattern-related proteins can be identified across studies and are relevant to chronic disease risk. However, future studies should use proteomic platforms with wider coverage and consistent data-analysis approaches to more comprehensively identify potential protein mediators.

## Potential Limitations of Proteomic Approaches in Nutrition Research

The proposed proteomic-based RCT-MR analysis has several potential limitations. First, proteomics platforms may not measure proteins accurately. To minimize measurement error due to preanalytical variation, biospecimens should be collected under standardized conditions for fasting status, time of day, interval since the last meal, sample type, processing time and temperature, storage, and freeze-thaw history [56], [57]. Although circulating levels are reasonably stable over time for a large majority of proteins, within-person variability in protein levels should be considered in the interpretation of the results [58]. Accurate measurement of protein affinity using affinity-based technology relies on conserved protein structures, but genetic variants can modify these structures. *cis-*pQTLs may include genetic variants that do not truly affect protein abundance but alter protein measurements by modifying protein structure at binding sites, referred to as “epitope effects” [12]. Results from a mass spectrometry-based proteomics study suggested that up to one-third of pQTLs were false positives due to epitope effects [59]. The likelihood of epitope effects can be assessed by evaluating whether pQTLs or variants in high linkage disequilibrium are protein-altering variants with biological effects that plausibly result in epitope effects [59]. Bias due to epitope effects could be addressed by confirming pQTLs using different proteomic platforms [59]. If both SomaScan and Olink identify pQTLs, they are less likely to reflect epitope effects. However, mass spectrometry confirmation is preferable, as this technique is not affected by affinity binding. In addition, lower accuracy or reproducibility may affect proteomic measurements, particularly for low-abundance proteins [14], [60].

Second, the assumptions of MR should be carefully considered [10]. For example, the genetic instruments used should be robustly associated with the exposure. Furthermore, horizontal pleiotropy is a concern, in which genetic variants affect biological pathways unrelated to the protein of interest. *cis-*pQTLs are less likely to affect other relevant biological pathways than trans-pQTLs and can be prioritized for use in MR. In addition, sensitivity analyses should evaluate the robustness of the results [28]. For example, the consistency of MR results across the genetic variants used can be evaluated. In addition, colocalization analysis can assess whether protein and disease associations share the same underlying causal variant within a gene locus. The value of the MR analysis for different diseases depends on the quality and size of available outcome GWAS. Large numbers of well-characterized cases increase statistical power, whereas disease heterogeneity and outcome misclassification can dilute associations and complicate causal interpretation [28], [61].

Third, proteins targeted by existing proteomic platforms (i.e., Olink and SomaScan) are enriched for secreted and abundant proteins. Furthermore, these proteomic assays quantify total circulating abundance rather than specific proteoforms, even though biologic activity may depend on isoform structure, cleavage state, or post-translational modification. In addition, proteomic measurements are primarily performed on blood samples. Diet-induced changes in blood protein levels may not reflect changes in protein levels in other disease-relevant tissues. For example, changes in brain or cerebrospinal fluid (CSF) protein levels are likely to be implicated in the pathophysiology of Alzheimer’s disease [62]. Randomized trials that collect CSF could examine the impact of dietary changes on CSF protein levels. If this is not feasible, comparison of protein levels across plasma, CSF, and brain tissue can be informative [63], [64]. In addition, cross-tissue pQTL comparisons and MR analyses of protein levels across tissues can distinguish whether effects are unique to blood, unique to other tissues, or shared [65].

Fourth, consistency for diet-proteomics results for trials and cohort studies may be reduced if the dietary pattern tested in the trial is not fully comparable to the dietary pattern score used in cohorts. More work is needed to develop closely related dietary pattern scores. An additional consideration is the use of relatively short-term feeding trials. Dietary factors can alter traditional risk factors, such as serum cholesterol and blood pressure, within weeks, and these changes can persist if dietary changes are maintained [66], [67]. However, it is uncertain whether changes in protein levels observed in short-term trials persist over the long term. Longitudinal observational studies can evaluate whether dietary changes are associated with long-term changes in protein levels. Regarding gene–environment equivalence [28], MR results reflect lifelong genetically induced differences in protein levels, which could be comparable to diet-induced protein differences if these reflect dietary patterns initiated in childhood. However, if disease processes become partly irreversible or if specific exposure periods are particularly important for disease development (e.g., early life or adolescence), lifelong genetically influenced differences in protein levels may not reflect the effects of dietary changes initiated later in life [28]. Finally, the proposed analyses can provide evidence for the beneficial effects of dietary patterns on chronic disease risk via selected circulating proteins. However, these findings cannot conclusively be interpreted as evidence for an overall beneficial effect of the dietary pattern, as other unknown mediators may act in the opposite direction. Similarly, proteomic-based analyses cannot readily estimate the total impact of dietary patterns on chronic disease outcomes, which is important for prioritizing dietary recommendations.

In terms of generalizability, diet-induced proteomic responses may differ across populations because of differences in baseline diet, the specific foods included in dietary patterns, physiological characteristics, and environmental factors. Still, we identified consistent associations between dietary patterns and protein levels across geographically and ethnically diverse populations [21]. Population differences in genetic architecture may affect MR that links proteins to disease outcomes. Allele frequencies and linkage-disease-equilibrium patterns differ across ancestry groups, and pQTL effect estimates and instrument strength may therefore not be transferable across populations [68]. Ideally, pQTL and outcome GWAS data should therefore be matched for ancestry.

## Comparison with Other Approaches Using Genetic Instruments in Nutrition Research

Another MR-based approach is to directly use genetic instruments as proxies for dietary intake. However, genetic proxies are unlikely to be identified for most dietary intakes. Suitable genetic instruments for food consumption would need to yield signals that individuals can detect and, in turn, affect dietary behaviors. For example, dairy intake is lower among individuals with a genetic predisposition to lactose intolerance because they lack the lactase enzyme in adulthood. Thus, these genetic variants can serve as a proxy for studying dairy consumption and disease risk [69]. One caveat is that fermented dairy products, such as yogurt, which may be particularly beneficial, are not well reflected in these genetic variants because they are low in lactose. Another example is coffee intake, for which the strongest genetic determinants affect the function of the liver enzyme that metabolizes caffeine [70]. Genetic proxies for coffee consumption habits may reflect caffeine metabolism, complicating the interpretation of MR results [71]. With careful interpretation, a limited number of dietary exposures can be directly captured by genetic proxies and studied using MR. However, many relevant dietary exposures, including dietary patterns, cannot be studied in this manner.

MR studies can also use genetic variants as instruments for metabolomic biomarkers of nutrition. For example, a variant in the methylenetetrahydrofolate reductase gene affects the availability of active folate and is associated with risk of neural tube defects, supporting the role of folate status in this condition [72]. MR studies of PUFA status in relation to cardiovascular diseases have yielded inconsistent results [73]. Although variants in fatty acid desaturase genes are substantially associated with PUFAs, these variants affect a range of fatty acids, making it difficult to attribute disease associations to a specific fatty acid. This illustrates that genetic variants associated with metabolomics markers may affect other biological pathways, potentially leading to horizontal pleiotropy [74]. Because genes code for proteins, *cis-*pQTLs may be more likely to be specifically associated with circulating protein concentrations. Still, metabolomic biomarkers may be well-suited to specific research questions if appropriate genetic instruments can be identified and MR assumptions are met.

Unlike selected metabolomic biomarkers [75], proteomic markers are unlikely to be specific biomarkers of dietary intake. Proteomic markers reflect the downstream effects of dietary exposures on biological processes rather than the exposure to specific dietary components. This is illustrated by the findings from the United Kingdom Biobank, in which several proteomic biomarkers were associated with multiple dietary patterns [17]. Thus, proteomic markers can be classified as intermediary or disease biomarkers rather than direct markers of dietary intake. A potential advantage is that proteomic markers identified as mediators of the impact of a dietary exposure on disease outcomes can be used to identify alternative interventions targeting these protein targets. For example, alternative dietary patterns that are more culturally appropriate for a population could be identified and shown to have similar effects on proteomic profiles. In addition, other lifestyle or pharmaceutical interventions may improve the protein targets.

In conclusion, in this paper, we describe how proteomics can be used in research on dietary patterns and chronic disease risk to inform causal inference. Protein biomarkers appear particularly useful for this purpose because they lie at the interface between genetic and environmental determinants and are involved in multiple biological pathways that contribute to chronic disease development. This facilitates their use as biomarkers of disease risk in randomized feeding trials and MR studies to elucidate their relevance to disease outcomes. Still, other biomarkers, such as selected metabolomic markers, may also be useful for specific research questions if suitable genetic instruments can be identified. We focused on dietary patterns and T2D as an example, but the same methodological approach can be applied to other dietary and lifestyle exposures and other chronic diseases. The value of the proposed approach for chronic disease outcomes will depend on several factors related to the limitations discussed in Potential limitations of proteomic approaches in nutrition research section. The framework may be particularly useful for diseases with long induction periods, such as cancer and neurodegenerative diseases, for which randomized dietary trials with clinical disease endpoints are often impractical. Furthermore, because most proteomic studies to date have relied on blood-based measurements, the availability of proteomic data from tissues relevant to the disease of interest may affect the biological interpretation and applicability of findings. Finally, the approach depends on the availability of sufficiently large outcome GWAS with accurate and consistent disease ascertainment, which remains limited for some chronic disease outcomes.

We highlighted potential limitations of the proposed method and areas for further research. For complex exposures such as diet, triangulating evidence across different research types is essential to strengthen causal inference [9]. Thus, the proposed RCT-MR approach should not be used in isolation but may be a useful addition to the nutritional epidemiology toolkit. Ultimately, methods that integrate multiomics data into nutrition research can contribute to more specific and robust dietary recommendations for chronic disease prevention.

## Author contributions

The authors’ responsibilities were as follows – RMvD, MCC: designed the research; RMvD, CGYL: reviewed the literature: RMvD: drafted the manuscript; MCC, CGYL: revised the manuscript; RMvD: took primary responsibility for the final content; and all authors: read and approved the final manuscript.

## Declaration of generative AI and AI-assisted technologies in the writing process

During the preparation of this work, the authors used Claude Opus 4.8 in order to improve the readability of sections drafted by the authors. After using this tool, the authors reviewed and edited the content as needed and take full responsibility for the content of the publication.

## Funding

This work was supported by a grant from the National Institute on Aging (grant number: R01AG065398).

## Conflict of interest

The authors report no conflicts of interest.
