# Supplementary material for: Dietary Patterns and Chronic Disease Risk: A Proteomic Framework to Strengthen Causal Inference
Source: Adv Nutr. 2026 Oct 13;17(12):100755. doi: 10.1016/j.advnut.2026.100755 (PMC13755564; doi:10.1016/j.advnut.2026.100755)
Supplement: Supplementary file 1 [file mmc1.docx]

**Supplementary Materials**

Title: Dietary Patterns and Chronic Disease Risk: A Proteomic Framework to Strengthen Causal Inference

Authors: Rob M. van Dam, Charlie G.Y. Lim, Marilyn C. Cornelis

| **Content** | **Page** |
| --- | --- |
| 1. Supplementary Table 1. Overview of proteomics studies on the DASH dietary pattern. | 2 |
| 2. Supplementary Table 2. Orthogonal validation of DASH-associated proteins measured on the SomaScan platform by correlation (r) with Olink measurements, existence of cis-protein Quantitative Trait Loci (cis-pQTL), and confirmation with mass spectrometry (MS). | 3 |
| 3. Supplementary Figure 1. Summary of associations between the DASH diet and blood protein concentrations. | 4 |
| 4. References | 5 |

**Supplementary Table 1.** Overview of proteomics studies on the DASH dietary pattern.

| **Author** | **Study name** | **Country** | **Ethnicity** | **Discovery and validation** | **Sample size** | **Dietary assessment** | **Proteomics platform** | **N proteins measured** | **N DASH-associated proteins** |
| --- | --- | --- | --- | --- | --- | --- | --- | --- | --- |
| Kim et al., 2023 (1) | DASH trial | USA | White, Black, other | Discovery | 215 | Controlled feeding | SomaScan | 7,241 | 71 |
| Kim et al., 2023 (1) | DASH‐Sodium trial | USA | Black, White, Asian, other | Discovery | 396 | Controlled feeding | SomaScan | 7,241 | 71 |
| Kim et al., 2023 (1) | Atherosclerosis Risk in Communities Study | USA | White and Black | Validation | 10,490 | FFQ | SomaScan | 5,284 | 71 |
| Zhu et al., 2025 (2) | UK Biobank | UK | Predominantly White | Discovery | 21,892 | 24-hour diet assessment | Olink | 2,915 | 852 |
| Lim et al., 2025 (3) | Jackson Heart Study | USA | Black | Discovery | 1,878 | FFQ | SomaScan | 1,317 | 60 |
| Lim et al., 2025 (3) | Singapore Multi-Ethnic Cohort | Singapore | Asian (Chinese, Malay, and Indian) | Validation | 2,395 | FFQ | SomaScan | 4,775 | 60 |
| Du et al., 2023 (4) | Atherosclerosis Risk in Communities Study | USA | White and Black | Discovery | 10,490 | FFQ | SomaScan | 4,955 | 254 |
| Du et al., 2023 (4) | Framingham Heart Study | USA | Predominantly White | Validation | 2,247 | FFQ | SomaScan | 1,373 | 254 |
| Sullivan et al., 2025 (5) | Chronic Renal Insufficiency Cohort | USA | White, Black, other | Discovery and validation | 2,217 | FFQ | SomaScan | 5,284 | 173 |
| Lee et al., 2026 (6) | Framingham Heart Study | USA | Predominantly White | Discovery | 740 | FFQ | Olink | 2,651 | 20 |
| Walker et al., 2020 (7) | Framingham Heart Study | USA | Predominantly White | Discovery and validation | 1,713 | FFQ | SomaScan | 1,373 | 83 |

**Supplementary Table 2.** Orthogonal validation of DASH-associated proteins measured on the SomaScan platform by correlation (r) with Olink measurements, existence of cis-protein Quantitative Trait Loci (cis-pQTL), and confirmation with mass spectrometry (MS).

|  | Katz et al., 2022 (8) | | Eldjarn et al., 2023 (9) | | Emilsson et al., 2018 (10) | | Wang et al., 2025 (11) | | Nicholas et al., 2026 (12) | | Summary across studies | | |
| --- | --- | --- | --- | --- | --- | --- | --- | --- | --- | --- | --- | --- | --- |
| Gene Symbol | r Soma-Olink | cis-pQTL SomaScan | r Soma-Olink | cis-pQTL SomaScan | MS confirmed | cis-pQTL SomaScan | r_s_ Soma-Olink* | cis-pQTL SomaScan | r Soma-Olink | cis-pQTL SomaScan | SomaScan cis-pQTL in any study | Soma-Olink correlation ≥ 0.50 | Cis-pQTL, high correlation, MS confirmed |
| EGFR | 0.50 |  | 0.46 | Yes | Yes |  | 0.57 | Yes | 0.46 |  | Yes | Yes | Yes |
| AFM |  | Yes | 0.61 | Yes | Yes | Yes | 0.69 | Yes | 0.63 | Yes | Yes | Yes | Yes |
| INHBC |  |  | 0.64 | Yes | Yes | Yes | 0.78 | Yes | 0.67 | Yes | Yes | Yes | Yes |
| MET | 0.61 | Yes | 0.51 | Yes | Yes | Yes | 0.75 | Yes | 0.58 | Yes | Yes | Yes | Yes |
| CCL25 | 0.58 | Yes | 0.37 | Yes |  | Yes | 0.49 | Yes | 0.50 | Yes | Yes | Yes |  |
| CNDP1 | 0.78 | Yes | 0.63 | Yes |  | Yes | 0.87 | Yes | 0.79 | Yes | Yes | Yes |  |
| INHBA |  |  |  | Yes | Yes |  |  |  |  |  | Yes |  |  |
| RBP4 |  |  |  | Yes | Yes | Yes |  |  |  |  | Yes |  |  |

*Spearman rank correlation; other correlations are Pearson correlations.

**Supplementary Figure 1**. Summary of associations between the DASH diet and blood protein concentrations.

All proteins were significantly and directionally consistently associated with the DASH dietary pattern in at least 4 studies.

Cells shaded in green (+) indicate a direct association between the DASH dietary pattern and the protein. Cells shaded in red (-) indicate an inverse association between the DASH dietary pattern and the protein. Cells shaded in grey (NS) indicate that there was no significant association between the DASH dietary pattern and the protein. Unshaded empty cells indicate that summary statistics for the protein were not reported in the study.

The proteomics studies included the UK Biobank (UKB) (2), Atherosclerosis Risk in Communities (ARIC) (4), Jackson Heart Study (JHS) (3), Singapore Multi-ethnic Cohort (MEC) (3), Chronic Renal Insufficiency Cohort (CRIC) (5), the Framingham Heart Study using SomaScan [FHS (Soma)] (7) and Olink Explore [FHS (Olink)] (6) and a meta-analysis of the results from the DASH trial and the DASH‐Sodium trial (DT-DST) (1).

**References**

1.	Kim H, Lichtenstein AH, Ganz P, Du S, Tang O, Yu B, et al. Identification of Protein Biomarkers of the Dietary Approaches to Stop Hypertension Diet in Randomized Feeding Studies and Validation in an Observational Study. J Am Heart Assoc. 2023 Apr 4;12(7):e028821.

2.	Zhu K, Li R, Yao P, Yu H, Pan A, Manson JE, et al. Proteomic signatures of healthy dietary patterns are associated with lower risks of major chronic diseases and mortality. Nat Food. 2025 Jan;6(1):47–57.

3.	Lim CG, Gradinariu V, Liang Y, Rebholz CM, Talegawkar S, Temprosa M, et al. Proteomic analysis identifies novel biological pathways that may link dietary quality to type 2 diabetes risk: evidence from African American and Asian cohorts. Am J Clin Nutr. 2025 Jan;121(1):100–10.

4.	Du S, Chen J, Kim H, Walker ME, Lichtenstein AH, Chatterjee N, et al. Plasma Protein Biomarkers of Healthy Dietary Patterns: Results from the Atherosclerosis Risk in Communities Study and the Framingham Heart Study. J Nutr. 2023 Jan;153(1):34–46.

5.	Sullivan VK, Chen J, Appel LJ, Schrauben S, Ricardo AC, Rao P, et al. Proteomic Markers of Dietary Patterns, CKD Progression, and Mortality in the CRIC Study. Clin J Am Soc Nephrol CJASN. 2025 Dec 1.

6.	Lee S, Joehanes R, Huan T, Liu C, Levy D, Ma J. Proteomics networks linking diet to cardiometabolic risk factors: the Framingham Heart Study. Am J Clin Nutr. 2026 Feb;123(2):101131.

7.	Walker ME, Song RJ, Xu X, Gerszten RE, Ngo D, Clish CB, et al. Proteomic and Metabolomic Correlates of Healthy Dietary Patterns: The Framingham Heart Study. Nutrients. 2020 May 19;12(5):1476.

8.	Katz DH, Robbins JM, Deng S, Tahir UA, Bick AG, Pampana A, et al. Proteomic profiling platforms head to head: Leveraging genetics and clinical traits to compare aptamer- and antibody-based methods. Sci Adv. 2022 Aug 19;8(33):eabm5164.

9.	Eldjarn GH, Ferkingstad E, Lund SH, Helgason H, Magnusson OT, Gunnarsdottir K, et al. Large-scale plasma proteomics comparisons through genetics and disease associations. Nature. 2023 Oct;622(7982):348–58.

10.	Emilsson V, Ilkov M, Lamb JR, Finkel N, Gudmundsson EF, Pitts R, et al. Co-regulatory networks of human serum proteins link genetics to disease. Science. 2018 Aug 24;361(6404):769–73.

11.	Wang B, Pozarickij A, Mazidi M, Wright N, Yao P, Said S, et al. Comparative studies of 2168 plasma proteins measured by two affinity-based platforms in 4000 Chinese adults. Nat Commun. 2025 Feb 21;16(1):1869.

12.	Nicholas JC, Katz DH, Tahir UA, Debban CL, Aguet F, Blackwell T, et al. Cross-ancestry comparison of aptamer and antibody protein measures. Nat Commun. 2026 Jan 22;17(1):1054.
